# Examining the perceptions and permissions of reusing treated wastewater in a region facing water scarcity

**DOI:** 10.1038/s41598-025-24308-w

**Published:** 2025-11-18

**Authors:** Hamza T. AL-Rikabi, Salah L. Zubaidi, Sandra Ortega-Martorell, Nadhir Al-Ansari, Nabeel Saleem Saad Al-Bdairi, Yousif Raad Muhsen, Khalid S. Hashim

**Affiliations:** 1 Department of Civil Engineering, Wasit University, Wasit, 52001 Iraq; 2 College of Engineering, University of Warith Al-Anbiyaa, Karbala, 56001 Iraq; 3 Data Science Research Centre, Liverpool John Moores University, Liverpool, L3 3AF UK; 4 Department of Civil Environmental and Natural Resources Engineering, Lulea University of Technology, Lulea, 971 87 Sweden; 5 Technical Engineering College, Al-Ayen University, Thi-Qar, 64001 Iraq; 6 Environmental Research and Studies Center, University of Babylon, Al‑Hillah, 51001 Iraq; 7 School of Civil Engineering and Built Environment, Liverpool John Moores University, Liverpool, UK

**Keywords:** Wastewater reuse, Public perception, SDGs, Survey, Sustainability, Iraq, Environmental sciences, Environmental social sciences

## Abstract

This study aimed to evaluate the public’s perspective on the utilisation of reclaimed water in Al-Kut, Iraq. Accordingly, a survey consisting of multiple-choice questions was designed to gather demographic information and assess the participants’ views and knowledge about water resources and wastewater reuse. The replies of 507 Iraqis, consisting of 326 males and 181 females, were analysed using a T, one-way analysis of variance (ANOVA), and Chi-square tests. The study’s findings indicate that 90.6% of the people are conscious of the issue of water crisis problem, whereas the others 9.4% either deny its existence (6.6%) or are uncertain about it (2.8%). Based on the survey questions, a high percentage (about 41%) of respondents have no idea that there is a water crisis. Nevertheless, the majority (88%) of individuals are implementing strategies to preserve water and are open to incurring additional charges for the installation of centralised wastewater treatment plants in the area. Many individuals support the utilisation of reclaimed water for agricultural and industrial applications. The findings also indicated a statistically significant relationship between sex, income, and acceptance of wastewater reuse. Incentives for reusing treated wastewater and opposition against it were ranked by the respondents.

**Supplementary Information:**

The online version contains supplementary material available at 10.1038/s41598-025-24308-w.

## Introduction

 The World Health Organisation and the United Nations Children’s Fund Joint Monitoring Programme for Water Supply and Sanitation have determined that 1.8 billion people around the world are likely to consume water that is contaminated with human waste^1^. As suggested by Roson and Damania^2^, the global accessibility of freshwater suitable for direct human consumption comprises a mere fraction, amounting to only 1/100th of the world’s total freshwater reserves, which represent just 3% of the total water supply. This stark reality underscores the significant mismatch between available resources and the burgeoning demand from human populations. By 2030, it is projected that the number of individuals experiencing serious shortages of water will increase twofold, reaching around 1.6 billion people, which accounts for over a quarter of the global population. Multiple investigations, for example, Smith, et al.^3^, Michetti, et al.^4^, and Mu’azu, et al.^5^, have indicated that urbanisation, industrialisation, water pollution caused by people, rapid population increase, and overuse of freshwater resources are all factors that contribute to the increase of water stress. Hence, effective and sustainable strategies to address the increasing demand for freshwater necessitate the implementation of appropriate methodologies and proactive measures by the relevant parties.

Water scarcity is a real problem, and one way to fix it is to supplement existing supplies with water from unconventional sources^6^. Wastewater recycling emerges as a promising solution to global freshwater challenges, as evidenced by its adoption by numerous nations^7–9^. By reducing sewage disposal, recovering freshwater from wastewater, and reusing it for municipal applications, this sustainable waste management strategy significantly contributes to the circular economy (CE) goals of resource recovery, zero waste, and reducing environmental pollution^10–12^. Recycled water is regarded as a viable alternative because research studies have demonstrated that wastewater may be sufficiently treated to ensure its safety for consumption^13–15^. Governments’ consideration of wastewater reuse reflects a strategic approach to bolstering freshwater resources^16^. Yue, et al.^17^ stated that wastewater reuse (WWR) is currently recognised as an essential element of Integrated Water Resources Management (IWRM). WWR is of utmost importance for several areas, including agriculture, industry, urban development, domestic reuse, potable water supply, and others, as it has been emphasised by several studies^16^.

Insufficient freshwater is a prevalent issue in nine Middle Eastern countries, including Iraq^18^. Furthermore, Iraq, a developing nation located in a semi-arid to dry region, is one of the Middle Eastern countries most vulnerable to the impacts of climate change^19^. Several causes, including global warming, the oil sector growth, increasing urbanisation, and the region’s already rapid population expansion, contribute to the region’s existing water scarcity^20^. The majority of Iraq’s freshwater is provided by the Euphrates and Tigris Rivers, where many storage dams have been constructed^21^. From 2009 to 2014, there was a severe water shortage in both rivers. The issue at hand will get worse due to climate change and the escalating water requirements from upstream nations such as Syria, Iran, and Turkey^22^. Salman, et al.^23^ projected multiple methods and scenarios indicating a probable rise in temperatures across Iraq, alongside a negative impact on future rainfall in terms of both pattern and quantity^24^. Furthermore, Ewaid, et al.^25^ highlighted that several studies have examined the quality of freshwater in Iraq, revealing an increase in various contaminants^26–28^. In addition, the water management of the rivers had adverse consequences following the terrorist attacks on many barrages and dams in Iraq after 2003^29^.

Iraq is thus facing more and more challenges in controlling its water supply. All too often, Iraq is now experiencing months-long droughts. Iraq is the thirty-ninth most water-stressed country and the fifth most vulnerable to climate change as a result of rising temperatures, inadequate and declining rainfall, long droughts, and water shortages^30^. Therefore, these challenges would directly threaten the water and food security systems (i.e., drinking water and agriculture). Worldwide, agriculture uses around 69% of the freshwater supply, mostly for irrigation, according to the UN Water Development Report^31^. There are already significant environmental, social, and economic impacts of climate change in Iraq, and the situation is getting worse as a result of farmers’ reliance on antiquated methods of water management and irrigation^32,33^.

The research will be focused on Al-Kut City, which is the capital of Wasit Governorate. For the most part, the freshwater supply is provided by the Tigris River and its branches. Unfortunately, there has been a 30% decline in the flow of the Tigris and Euphrates rivers since the 1980s, affecting 98% of Iraq’s water supply^34^. Also, At the height of the drought in 2008, wheat output was 47% lower than the year before, according to the World Bank report in 2018^33^. One of the governorates’ claims to fame is its pioneering work in growing strategic crops; when it comes to vital crops, like wheat, Wasit is Iraq’s food basket. The high rate of agricultural use depletes water resources. There is a severe water crisis due to its dependence on the Tigris River, which suffers from water scarcity. Also, Wasit is feeling the pinch of climate change in the form of rising temperatures and less precipitation. As per the data provided by the Ministry of Municipalities’ Department of Sewerage, the City of Kut generates around 65,000 cubic metres of wastewater every day. Moreover, the government plans to establish central wastewater treatment plants in the region. All of these indicators confirm that water security in Wasit Governorates is extremely fragile and requires urgent, sustainable solutions. Wastewater reuse is proposed as one such solution to alleviate water scarcity and improve water sustainability.

Worldwide, the topic of wastewater reuse has been extensively discussed^35,36^, and the degree of its acceptance varies widely. As an example, Al-Khatib, et al.^37^ noted that there is a correlation between factors such as education, age, income, and sex, and the level of acceptance for using recycled water. Accordingly, Abdelrahman, et al.^38^ studied the attitudes toward water reuse, and argued that emotions could facilitate the evaluation of complicated situations, significantly impacting both, attitude and behaviour. Attitudes can be evaluated via a model consisting of cognitive, behavioural, and emotional components. Cottrell^39^ utilised the three-component attitude model to gain a deeper understanding of how emotions influence attitudes and behaviours.

One potential barrier to the acceptance of wastewater reuse may be the deep-seated emotions that elicit negative attitudes toward water reuse^40^. Hence, it is crucial to get knowledge regarding the public’s attitudes toward the concept of recycling treated wastewater to understand the emotional responses and individual differences associated with this subject. Utilising this data, campaigns promoting wastewater recycling should employ more precise targeting strategies to effectively reach the general community. The success of wastewater reuse facility development, building, and operation relies heavily on the public’s attitudes, approval, and support. Various studies have investigated the contributing factor of public attitudes, encompassing variables such as sex, educational attainment, income level, cultural background, religious affiliation, health status, sources of information, and level of knowledge^41–43^.

Studies like^44–46^ indicate that both sexes find it acceptable to use treated wastewater for non-skin contact activities, such as flushing toilets, washing cars, and watering plants. Furthermore, according to Abdelrahman, et al.^38^, regardless of the level of treatment, a large number of people are opposed to using recycled wastewater. However, as the application shifts from direct physical touch to indirect nonphysical contact, the resistance decreases. Several prior studies conducted on nations neighbouring Iraq yielded varying results regarding the acceptance of treated water reuse. In two investigations from Oman, respondents opposed using treated wastewater for direct contact. The first survey found that 64.5% of respondents would reuse treated wastewater for gardening and 42.7% for car cleaning^47^. The second study found that 64.2–78.7% of attendees were optimistic about using treated wastewater to irrigate non-edible crops, business landscapes, fire hydrants, cool buildings, golf courses, public parks, school grounds, toilet flushing, and car washing^48^.

Moreover, another study from Palestine Al-Khatib, et al.^49^ found that most respondents opposed using treated wastewater for direct contact. However, aquifer recharge and aquaculture were more accepted—38% and 42%, respectively. In a study conducted in the United Arab Emirates, Abdelrahman, et al.^38^ found that a majority of the public (55%) expressed a preference for utilising treated wastewater for irrigating non-food crops. However, they tended to use it for irrigating food crops, with a rejection rate of 66%. According to another study, 76% of the public in the Muscat Governorate of Oman accepted the use of recycled wastewater for gardening, while 66% approved its use for toilet flushing and 53% approved its use for car washing^50^. A state-wide survey revealed that there was widespread concern among the population in Turkey regarding the possible health risks associated with the reuse of wastewater. Nevertheless, they consented to repurpose it for activities like flushing toilets and cleaning roads and buildings, which do not require direct interaction with humans^37^. Additional global investigations, such as Brazilian respondents Faria and Naval^51^, use treated wastewater for indirect applications (watering plants and gardens, washing clothing, and cleaning), 98% and 74% for complete and incomplete higher education, respectively. Also, Over 50% of Tanzanian respondents approved of using treated wastewater in diverse applications, while 93% were opposed if it included direct water contact^52^.

Furthermore, Li and Roy^53^ mentioned that although conservation efforts and informational campaigns can help change people’s minds about recycled water, many individuals are still unsure about how they feel about using it. Leong and Lebel^54^ reported that numerous research have revealed that a multitude of factors contribute to this mistrust. These aspects encompass sociodemographic variables, community involvement, awareness levels, water availability and access, health concerns, and the water’s intended usage. However, customers’ attitudes and intentions to buy potable reuse water should improve as their perceived knowledge or experience with water reuse increases^55^. Mu’azu, et al.^5^stated that previous studies did not confirm the relationship between higher education levels and acceptance of treated wastewater. Some large-scale research in wealthy countries found no statistically significant association. In poorer countries, reuse acceptance decreases with education. In contrast, different studies on treated wastewater reuse show that better education increases acceptance.

This research falls within the context of global efforts to achieve sustainable development, contributing to the Sustainable Development Goals (SDGs) adopted by the United Nations, particularly in the field of water resource management. The research examines the community’s acceptance of reused water, which is closely linked to SDG 6, “Clean Water and Sanitation,” which seeks to improve water management and reduce pollution through recycling and safe use of reused water. This research also supports SDG 11, “Sustainable Cities and Communities,” by providing solutions that enhance the sustainability of urban water resources, reducing pressure on freshwater and contributing to improved sanitation infrastructure. Furthermore, the research aligns with SDG 13, “Climate Action,” as reducing the consumption of freshwater resources through reuse can contribute to mitigating the effects of climate change and reducing the risk of drought. The research also supports the achievement of SDG 12, “Responsible Consumption and Production,” by encouraging recycling policies and sustainable resource use, reducing water waste, and promoting responsible environmental practices. Accordingly, the findings of this research can provide a scientific framework that helps decision-makers develop more sustainable water management strategies, in line with the global Sustainable Development Goals.

The primary aim of this study is to evaluate public acceptance and attitudes regarding using treated wastewater for different usages in Al-Kut City. So, the major objectives of this study were to: (1) Determine how well people understand the water scarcity issue. (2) Investigate how people of different ages, sexes, educational backgrounds, and income levels feel about using recycling wastewater for agricultural, commercial, and industrial purposes; and (3) Examine what motivates people to take advantage of wastewater reuse incentives and what stops them from expanding reclaimed water reuse.

## Research methodology

### Collection of data

A stated preference survey was developed and distributed to people in Al-Kut City, Iraq, to examine their attitudes and acceptance regarding the reuse of reclaimed water. This survey consists of 32 questions divided into five categories. The potential participants were randomly selected and they were initially asked about their willingness to be part of this study and answer the questions to ensure higher response accuracy. As such, the survey was only distributed to individuals who agreed to participate in this study. For the current study, we revised the questionnaire used by Abdelrahman, et al.^38^., incorporating new questions and removing the ones that are not relevant to Iraq (please see supplementary material).

Between December 15, 2023, and March 14, 2024, a continuous period of three months was dedicated to conducting a survey. The survey was conducted using both Google Forms and printed copies to target seniors and retirees who were not familiar with digital platforms. A stratified random sampling approach was used to ensure diversity across key demographic groups. Participants were randomly selected from different geographic areas of the city and across ethnic lines (e.g., Arab and Kurdish communities) to ensure adequate representation. The representative sample consisted of 507 participants out of 1,000 participants, yielding a response rate of 50.7%. The final response rate of 50.7% falls within the generally accepted range of 40–60% for social surveys, as noted by Baruch and Holtom^56^, and is considered adequate for drawing inferences about the target population. To ensure that the participants in the study accurately represented the study area, a minimum sample size was utilised. In their 2014 study, Dillman, et al.^57^ formulated an equation to determine the most favourable sample size, as shown in Eq. (1):

1 $$\:n=\frac{{z}^{2}\times\:p\times\:(1-p)}{{MoE}^{2}}$$

Where: n = full sample size required to achieve target precision, p = the percentage under examination, MoE = the target margin of error for the sample, z = critical value for the desired confidence level.

As the margin of error increases, the confidence level in the survey results decreases. Research that analyses survey data frequently uses a confidence level of 95%. Therefore, with a confidence level of 95%, the *z*-value is 1.96. Furthermore, the current investigation employs a conservative value of p that is evenly split 50/50. The margin of sampling error was ultimately established at 4.5. By substituting these numbers into Eq. (1), we find that a size of 475 is necessary for this inquiry. Therefore, the number of survey responses gathered for this inquiry, which was 507, surpassed the minimum requirement for a 95% confidence level.

### Steady of area

**Fig. 1:**
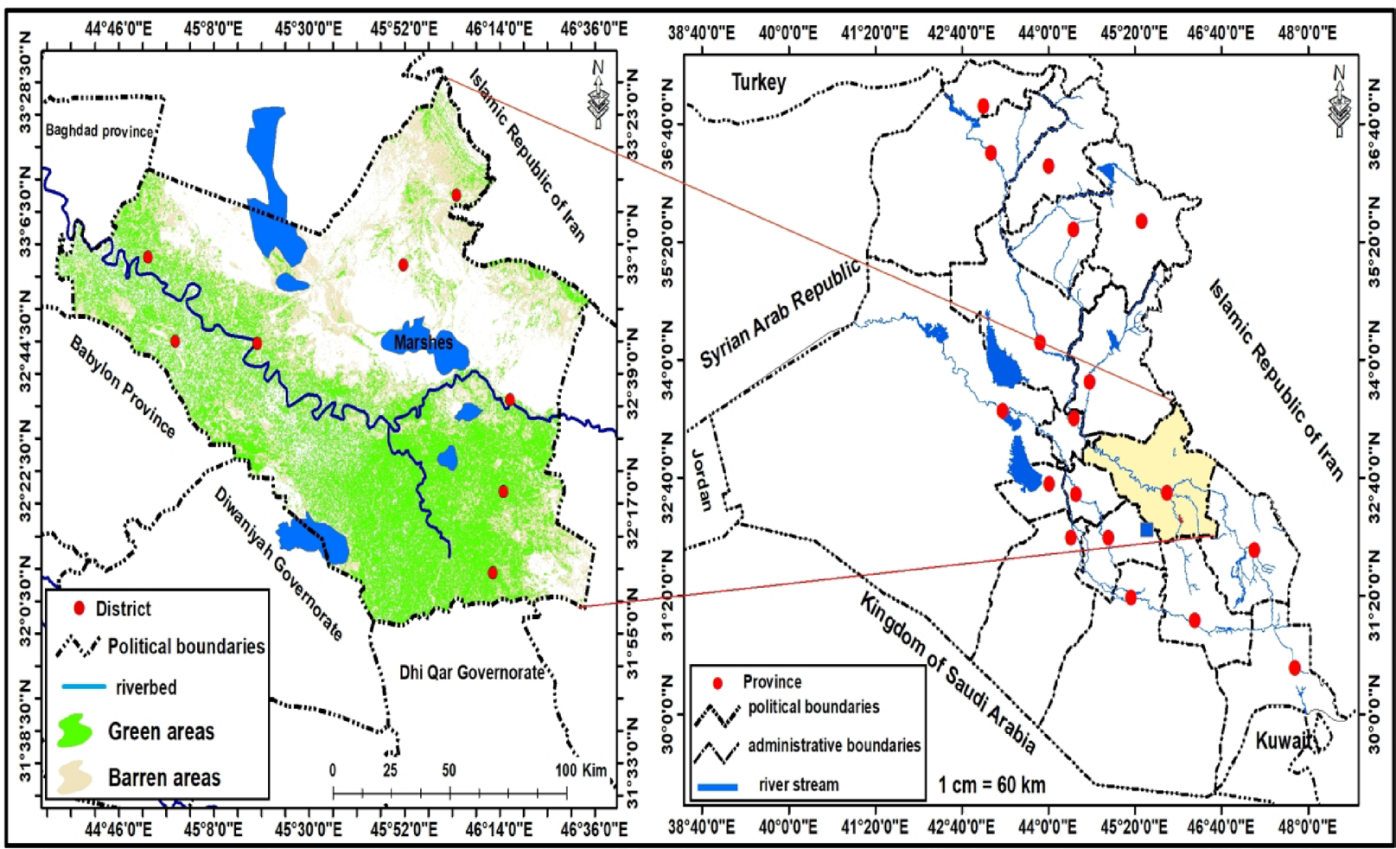
Location map of the research area (Wasit Governorate).

Al-Kut is located on the banks of the Tigris River in southern Iraq and is the capital of the Wasit Governorate^58^ (Fig. 1). The metropolis is vast, covering an area of 17,153 square kilometres. Conversely, the urban area of Al-Kut spans approximately 40 square kilometres. Forecasts suggest that the population of the city will increase significantly to more than 750,000 by 2035, compared to 400,000 in 2003. Al-Kut is renowned for its agricultural prowess in wheat production^59,60^. The mean altitude between two longitudes (45° 54^/^ and 45^o^ 45^/^) east and two latitudes (32^o^ 21^/^ and 32^o^ 34^/^) north is approximately 20 m^61^. The climate in Al-Kut City is characterised by agreeable seasons, with nice spring and autumn, frigid winters, and dry, hot summers. As per the Iraqi Meteorological Department, the winter season commences in November and lasts until March. The summer season spans the following seven months, often with June, July, and August experiencing their highest temperatures^60,62^.

### Study instrument

This study utilised a stated preference survey, as described in Sect. "Collection of data" and supplementary material. The first section of the survey (questions 1–14) asked participants to provide details about themselves, including their demographic and other personal details. It is mainly based on multiple-choice and binary (Yes/No) or closed-category (multiple-choice) questions. Moreover, the 2nd section, encompassing questions 15–21, evaluated the respondents’ attitudes and levels of knowledge of water resources and the recycling of wastewater. Except for question 18, it includes binary questions (Yes/No), and optional questions (such as the source of knowledge). Question 18, uses the Likert scale, where responders need to select from a range of multiple-choice options. Through the analysis of the data derived from these questions, we can evaluate its validity and dependability. Question 18 is designed to measure the extent to which individuals engage in water conservation practices in their households. On a three-point scale, you can choose between “always,” “sometimes,” and “never” to answer this question.

Section three (questions 22–26) assessed individuals’ attitudes towards the reuse of treated wastewater, the establishment of centralised wastewater treatment systems, and their confidence in the safety regulations for treated wastewater. It includes binary questions (Yes/No) like questions 23 and 26., and two Likert Scale types (5 points). Question 22 is designed on a five-point Likert Scale (from “I strongly agree” to “I strongly refuse”) to measure the extent to which individuals engage in using recycled wastewater for some purposes. Question 25 is designed on a five-point Likert Scale (from “Fully trust” to “Have no knowledge of it”) to measure the extent to which individuals trust the safety criteria of treated wastewater that you can use.

Questions 27–30 (fourth section) required participants to assess their level of confidence in different applications of treated wastewater, as well as their justifications for accepting this usage. It measures respondents’ confidence in the different applications of treated wastewater based on a four-point Likert Scale. Answers to these questions can be categorised as either “strongly support”, “support”, “do not support”, or “do not know” on a four-point scale. The fifth section, questions 31 and 32, assessed the participants’ views on the public’s perceptions of the benefits and obstacles of reusing treated wastewater. These questions are based on ranking questions, which differ from the Likert scale. The “First” option goes to the most important followed by other options for the least important.

Cronbach’s alpha, which can be expressed as a value between 0 and 1, was used to determine the questionnaire’s internal consistency. A test’s reliability might be defined as the degree to which it consistently yields the expected results. A test’s reliability can be expressed as a function of its item count and average inter-item correlation, which is known as Cronbach’s Alpha^16^. The reliability of the questionnaire was evaluated using the Statistical Package for Social Science SPSS, Version 29, software. As the reliability value approaches 1, it indicates a lower level of error. For instance, when the reliability is 0.9, it means that, on average, the variables are 90% accurate with a 10% margin of error. The formula for Cronbach’s alpha can be found in Eq. (2). A survey was given out to a sample of forty individuals to establish its reliability and validity. The questionnaire items 18, 27, 28, 29, and 30 were found to have reliability values of (α = 0.928) and validity of 0.963.

2 $$\:\propto\:=\frac{N\stackrel{-}{c}}{\stackrel{-}{\upsilon\:}+\left(N-1\right)\stackrel{-}{c}}$$

Where: α = Cronbach’s alpha, N = number of items, $\:\stackrel{-}{c}$= the average interitem covariance, and υ = average variance.

### Analysis of data

A statistical analysis was conducted to define the frequency distribution of the data and to test the study hypotheses. The SPSS, Version 29, was utilised for the preparation and testing process. The analysis of the participants’ characteristics was conducted using frequency analysis. This analysis included their sex, ages, incomes, and educational levels. The relationship between sex and the utilisation of treated wastewater for various purposes was analysed using the T-test. An analysis was conducted to examine the correlation between confidence in wastewater reuse for various purposes and factors such as age, level of education, and income. The analysis employed the ANOVA test. The Chi-square technique was applied to analyse participants’ viewpoints on the relative significance of several incentives for the public utilisation of reclaimed water, based on factors such as sex, age, education level, and income. An additional analysis was performed utilising the Chi-square technique to figure out the correlation between sex, age, education level, income, and the public’s assessment of the obstacles to reusing treated wastewater. Table 1 presents the demographic characteristics of the 507 respondents.

**Table 1:** Participants demographic information.

	Demographic information	*N*	Percent
1	*Sex*: Male Female	326 181	64.3 35.7
2	*Age*: 18–25 25–30 30–35 35–40 40–45 45–50 50–55 55–60 more than 60	103 152 101 50 37 25 23 11 5	20.3 30.0 19.9 9.9 7.3 4.9 4.5 2.2 1.0
3	*Marital Status* Married Unmarried Divorced Widowed/Widow	275 227 3 2	54.2 44.8 0.6 0.4
4	*Occupation*: Doctor Engineer Lawyer Government employee Teacher Private employer Student Other	26 62 9 183 51 73 65 38	5.1 12.2 1.8 36.1 10.1 14.4 12.8 7.5
5	*Qualification*: Primary Intermediate Secondary Diploma Undergraduate Postgraduate	18 20 80 82 216 91	3.6 3.9 15.8 16.2 42.6 17.9
6	*Is there a child living at home?* Yes No	397 110	78.3 21.7
7	*No. of children*: From 1 to 3 From 4 to 6 From 7 to 9 > 9	288 93 11 5	56.8 18.3 2.2 1.0
8	*Income*: From 300,000 to 600,000 Iraqi dinars (IQ) From 600,000 to 900,000 IQ From 900,000 to 1,200,000 IQ From 1,200,000 to 1,500,000 IQ More than 1,500,000 IQ	158 90 57 36 166	31.2 17.8 11.2 7.1 32.7
9	*Paying fees* Yes No	416 91	82.1 17.9
10	*Amount paying*: 5000–15,000 IQ 15,000–25,000 IQ 25,000–35,000 IQ 35,000–45,000 IQ Equal & Greater than 45,000 IQ	117 187 44 1 4	23.1 36.9 8.7 0.2 0.8
11	*Sewerage network connected*: Yes No	412 95	81.3 18.7

### Ethics statement

Before asking the participants in this study about their opinions, the purpose of the questionnaire was clearly explained, and they were asked about their willingness to participate in the survey. Accordingly, this study only asked participants who were willing to participate in the questionnaire. Plus, they were informed that written informed consent is required, and the anonymity of their responses is assured. As such, the study is completed only after receiving the assent from the participants. It is crucial to note that this study adhered to the ethical principles outlined in the Declaration of Helsinki, and all procedures for conducting the questionnaire that involved human participants were approved by the Institutional Review Board of the University of Wasit, Iraq.

## Results and discussion

Understanding public attitudes and acceptance of recycled water is crucial for the successful implementation of wastewater reuse initiatives^63^. Research studies have also linked general water or water treatment process knowledge to a higher rate of recycled water adoption, such as water for close-contact uses^64^. Extensive research suggested that individuals tend to be more receptive to using recycled water for drinking purposes when they exhibit a greater level of self-reported knowledge or awareness regarding the subject^65–67^. To identify subsets of the population displaying heightened enthusiasm for the reuse of treated wastewater compared to the general populace, Baghapour, et al.^43^ and Dolnicar and Schäfer^68^ conducted comparative research on public awareness, attitudes, and acceptance of the topic. According to their findings, treated wastewater is acceptable for nondirect contact usage, including industrial processes, irrigation of crops that are not food crops, and others.

### Knowledge

**Fig. 2:**
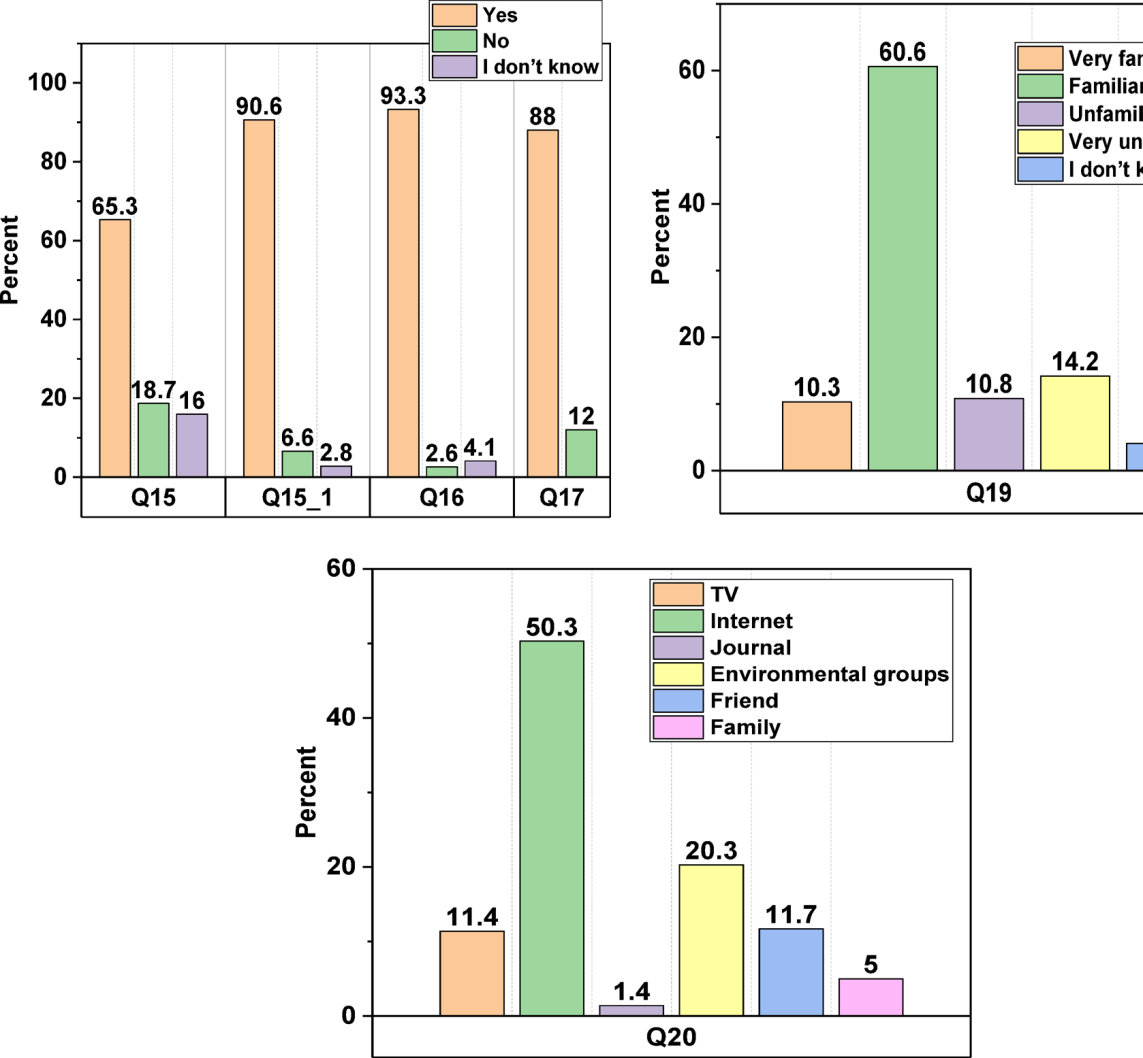
Participants’ responses to questions 15, 15 − 1, 16, 17, 19 and 20.

The response to question 15, querying, “Do you have any knowledge about the water available in your country?” emerges as the pivotal discovery of this study. As illustrated in Fig. 2, approximately 65.3% of respondents indicated awareness of water availability in Iraq, while 34.7% either expressed uncertainty (16%) or claimed no knowledge (18.7%). Notably, regarding question 15.1 (“If your response to question 15 is ‘yes’, In your opinion, does Iraq have an inadequate supply of natural water resources?“), further insights are warranted. The data depicted in the figure indicates that 90.6% of respondents selected “yes,” while the remaining 9.4% either answered “no” (6.6%) or expressed uncertainty (2.8%). Drawing from the preceding questions, it is notable that a considerable proportion (approximately 41%) of survey participants lack awareness of the water crisis and governmental efforts to ensure adequate water supply. This underscores a significant area of concern.

**Fig. 3:**
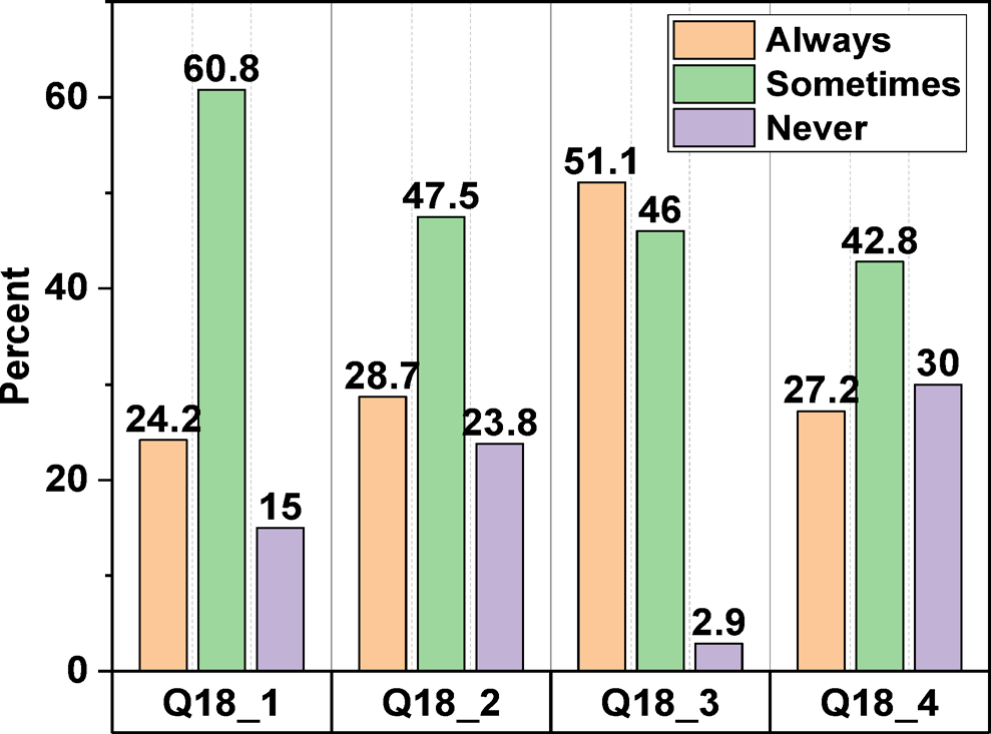
Participants’ response to question 18.

Regarding their answer to question 16, which inquired, " Is it necessary to reduce water consumption in Iraq, in your opinion?” a substantial majority (93.3%) of survey participants expressed support for water rationalisation. Furthermore, nearly nine out of ten respondents affirmed their commitment to conserving water consumption in their households in response to question 17. Question 18 subsequently prompted participants who responded affirmatively to question 17 to delineate the specific measures undertaken to conserve water in household applications. Figure 3 illustrates that respondents implemented water-saving practices such as reducing water usage during cleaning or bathing, transitioning to toilets with lower water consumption, and upgrading to water-saving taps. However, their responses varied, with percentages indicating “sometimes” ranging from 43% to 61%, while “never” responses ranged from 3% to 30%. Additionally, a notable finding is that participants expressed a need to know more about the country’s water conservation efforts and how they benefit people, the environment, and society as a whole.

Concerning question 19 (“Do you have any background knowledge on treated wastewater?“), the findings revealed that approximately 30% of survey participants had never encountered the concept of wastewater recycling. This underscores the importance of public education initiatives aimed at this demographic, emphasising the economic and ecological advantages associated with wastewater reuse. Regarding question 20 (“What sources have you used to learn about treated wastewater reuse?“), the primary sources cited were the Internet, followed by environmental groups as a distant second, as indicated in Fig. 2. Contributions from friends, television, family, and journals were comparatively less substantial. Likewise, Chfadi, et al.^69^ observed that the Internet serves as the predominant avenue for public information dissemination. As for question 21 (“From your different backgrounds, how would you suggest communication with the public be when implementing this type of project?“), respondents were afforded free choice. In efforts to enhance public attitudes toward wastewater recycling, they proposed various methods outlined in Table 2.

**Table 2:** Ways suggested to Raise consciousness about water reuse.

Suggested methods	Number	Percent
Social media	150	39%
Internet advertisements	60	16%
Awareness through the media	75	20%
By organising workshops or advisory meetings	33	9%
Field mobile team campaigns in the cities	29	8%
Spreading awareness in universities, schools, and mosques	10	3%
I don’t know	24	6%
Total	381	100%

The results in this section align with the study’s first objective, which aimed to assess the population’s awareness of water resources and their reuse. The limited awareness of water resource sustainability may be a significant obstacle to implementing water reuse policies. Therefore, increasing awareness campaigns, mainly through the media and social media platforms, may improve the population’s acceptance of these technologies and raise awareness of the importance of reuse. According to earlier research studies, the level of public awareness and knowledge is a key factor in the success of any recycling initiative^70,71^.

### Attitude

**Fig. 4:**
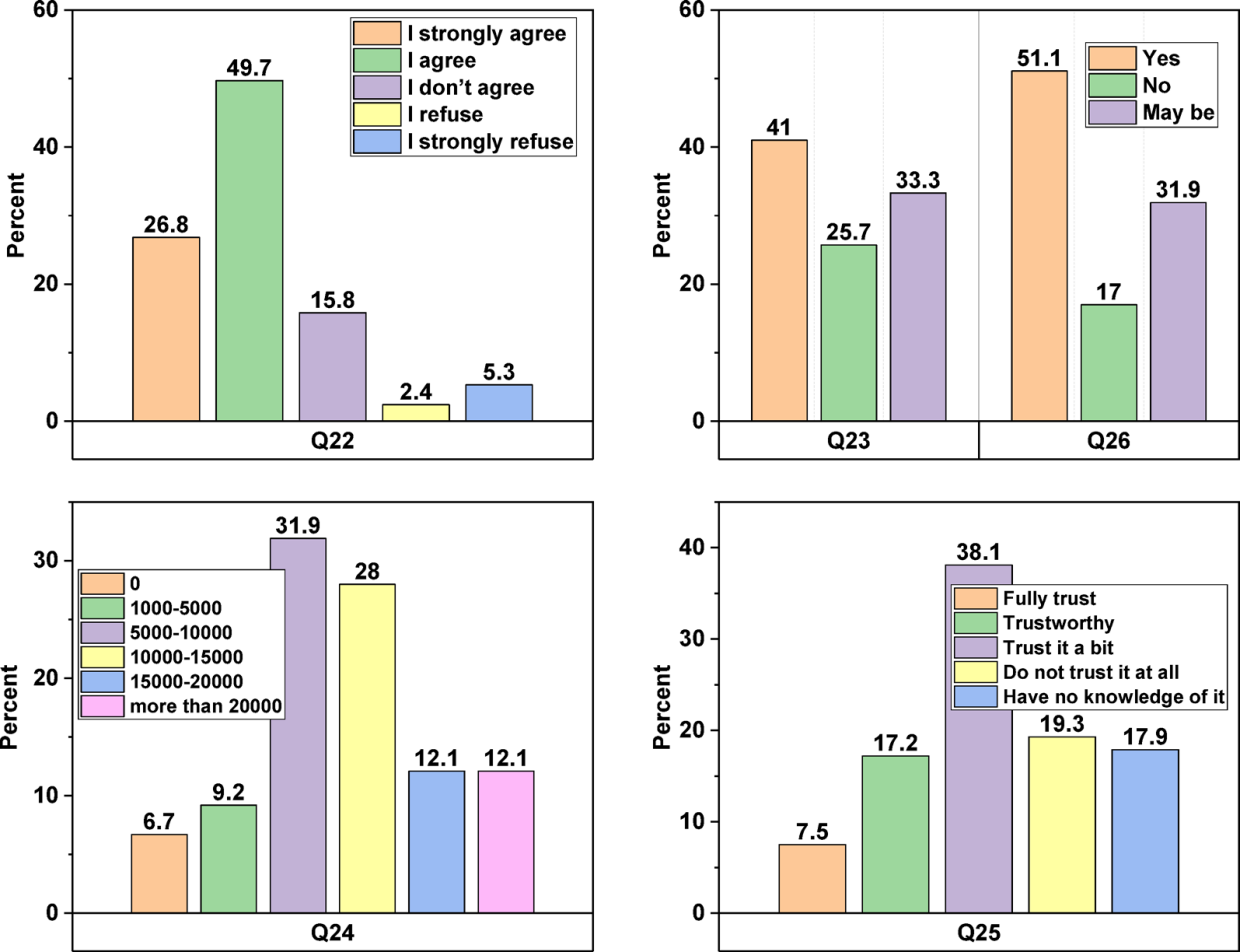
Participants’ responses to questions 22–26.

Baghapour, et al.^43^, Raman^72^, Buyukkamaci and Alkan^73^, Domènech and Saurí^74^, Kantanoleon, et al.^75^, DuBose^76^, Dolnicar, et al.^77^ and Maraqa and Ghoudi^78^, pointed out the public’s conditional approval of using reclaimed water. In question 22 (“Are you in favour of using recycled wastewater for some purposes?“), Fig. 4 distinctly delineates two groups, representing those in agreement and those in disagreement. The studies conducted by DuBose^76^, Aitken, et al.^79^, Browning-Aiken, et al.^80^, and Leviston, et al.^81^ forecasted substantial support for water-treated initiatives when crafted with sustainability as a central focus.

Contrary to previous studies, Chfadi, et al.^69^ and Baghapour, et al.^43^, found that the reclaimed water acceptance was highest when there was minimal skin contact and lowest when it was linked to activities such as drinking, cooking, washing, and bathing. Domènech and Saurí^74^ underscored that the public’s tendency to establish centralised wastewater treatment facilities in their homes is significantly shaped by considerations such as health risks, operational procedures, pricing, and environmental awareness, particularly in Spain. In our study, concerning question 23 (“Would you be open to having a central system installed in your home?“), approximately 75% of participants responded with either “yes” or “maybe.” Subsequently, question 24 inquired, “If your answer to question 23 is yes or maybe, how much extra monthly fees are you willing to pay?“. Approximately 60% of respondents indicated they would be willing to pay between 5,000 and 15,000 IQ, equivalent to around 4 to 12 US dollars, each month for the installation of a central wastewater treatment system, as depicted in Fig. 4. Conversely, in answer to the 25th question (“How much do you trust the safety standards for treated wastewater that you can use?“), Fig. 4 shows that more than 62% of Iraqis have faith in the safety rules put in place by the government.

Table 3 presents a summary of responses to questions 27 through 30. While 16.4% of respondents expressed opposition to using treated wastewater for watering food crops, a significant majority (70.4%) supported its use for non-food crops. On average, 16% of survey participants objected to using treated wastewater for irrigation, while 14.1% remained uncertain, as outlined in Table 3. The study conducted in Canada by Velasquez and Yanful^64^ found that 76% of participants endorsed using treated wastewater for irrigating food crops, while 86% supported its use for non-food crops. The adoption rate was relatively higher for low-contact applications such as the irrigation of public parks and animal crops compared to high-contact applications such as food crops irrigation. Menegaki, et al.^82^ emphasised the significance of attitudes in exploring both the usage and payment intentions for using reclaimed water in agriculture. According to Table 3, on average, a significant majority (66.2%) of respondents express support for treating wastewater for commercial and industrial purposes. This support extends to various applications, including power plants (69%), cooling, cleaning, and car wash averages (70%), construction operations (69%), firefighting (75%), and laundry services (43%). According to Table 3, a majority of respondents express support for recycling wastewater into storage for emergencies (61.5%) and artificial lakes (60.8%). However, they are against its usage in the following areas: cooking (59.6% ), cleaning produce (i.e., vegetables and fruits) (55%), watering pets and birds (24.5%), domestic usage (51.9%), swimming pools (48.7%), and fish farms (32.9%).

**Table 3:** A synopsis of the participants on questions concerning utilising recycled wastewater for agriculture, industry and commerce, other purposes and purpose of their suppor.

		Strongly support	Support	Don’t support	I don’t know	m	SD	Rank
**27-How will you trust using treated wastewater for irrigation**								
Irrigation of food crops	N	137	209	83	78	2.20	1.005	2
	%	27	41.2	16.4	15.4			
Irrigation of non-food crops	N	149	208	71	79	2.16	1.017	2
	%	29.4	41	14	15.6			
Irrigation of public parks	N	190	207	58	52	1.94	0.948	2
	%	37.5	40.8	11.4	10.3			
Animal crops	N	162	218	68	59	2.05	0.958	2
	%	32.0	43.0	13.4	11.6			
Irrigation All crops	N	124	182	112	89	2.33	1.031	2
	%	24.5	35.9	22.1	17.6			
Average		30.1%	40.38%	15.46%	14.1%	2.1357	0.9918	
**28- How confident with using treated wastewater for industry and commerce**								
Cooling (e.g. cooling of power plants - cooling machines ….)	N	178	189	60	80	2.08	1.047	2
	%	35.1	37.3	11.8	15.8			
Construction work (such as: construction work - mixing concrete…)	N	166	181	87	73	2.13	1.029	2
	%	32.7	35.7	17.2	14.4			
Power plants (e.g. steam production ………….)	N	158	191	73	85	2.17	1.049	2
	%	31.2	37.7	14.4	16.8			
Car wash	N	135	198	114	60	2.20	0.963	2
	%	26.6	39.1	22.5	11.8			
Clothes Laundries	N	67	150	219	71	2.58	0.889	3
	%	13.2	29.6	43.2	14			
Cleaning (street cleaning – cleaning)	N	160	198	87	62	2.10	0.984	2
	%	31.6	39.1	17.2	12.2			
Fire Fighting	N	187	190	44	86	2.06	1.064	2
	%	36.9	37.5	8.7	17			
Average		29.6%	36.6%	19.3%	14.6%	2.1879	1.0035	
**29- How confident with using treated wastewater for the following purposes**								
Artificial lakes	N	116	192	130	69	2.30	0.970	2
	%	22.9	37.9	25.6	13.6			
Swimming Pools	N	55	132	247	73	2.67	0.854	3
	%	10.8	26.0	48.7	14.4			
Fish Farms	N	84	180	167	76	2.46	0.939	2
	%	16.6	35.5	32.9	15.0			
Household uses (e.g. bathing ………….)	N	53	125	263	66	2.67	0.831	3
	%	10.5	24.7	51.9	13.0			
Storage for emergency	N	111	201	122	73	2.31	0.970	2
	%	21.9	39.6	24.1	14.4			
Drinking animals and birds	N	108	211	124	64	2.28	0.940	2
	%	21.3	41.6	24.5	12.6			
Washing vegetables and fruits	N	60	111	279	57	2.66	0.830	3
	%	11.8	21.9	55.0	11.2			
Cooking	N	44	98	302	63	2.76	0.779	3
	%	8.7	19.3	59.6	12.4			
Flushing toilet	N	145	234	75	53	2.07	0.921	2
	%	28.6	46.2	14.8	10.5			
Average		17%	32.5%	37.5%	13%	2.464	0.893	
**30- The extent to which you support the use of treated wastewater for achieving the following goals**								
Preserving the environment	N	248	187	30	42	1.74	0.901	1
	%	48.9	36.9	5.9	8.3			
Easing pressure on groundwater and high-cost desalinated water	N	221	218	24	44	1.79	0.886	2
	%	43.6	43.0	4.7	8.7			
Reducing pollution	N	246	200	17	44	1.72	0.889	1
	%	48.5	39.4	3.4	8.7			
Giving up the purchase of chemical fertilisers harmful to Public health	N	258	193	10	46	1.69	0.894	1
	%	50.8	38.1	2	9.1			
Average		47.95%	39.35%	4%	8.7%	1.735	0.893	

**Fig. 5:**
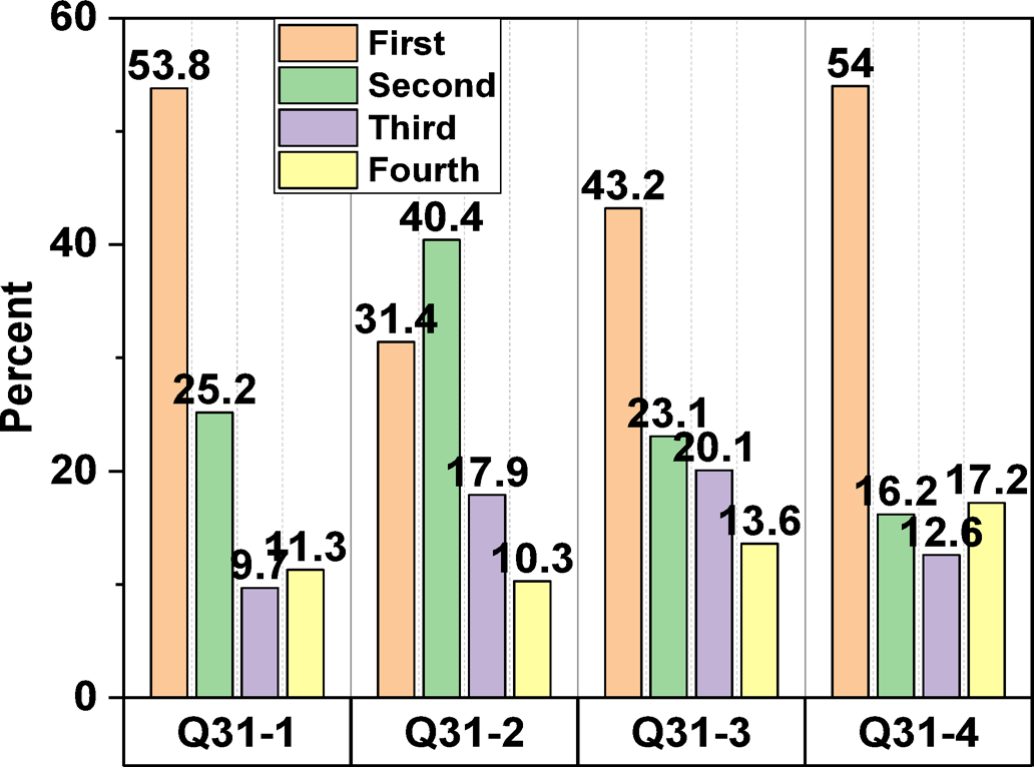
Participants’ response to question 31.

**Fig. 6:**
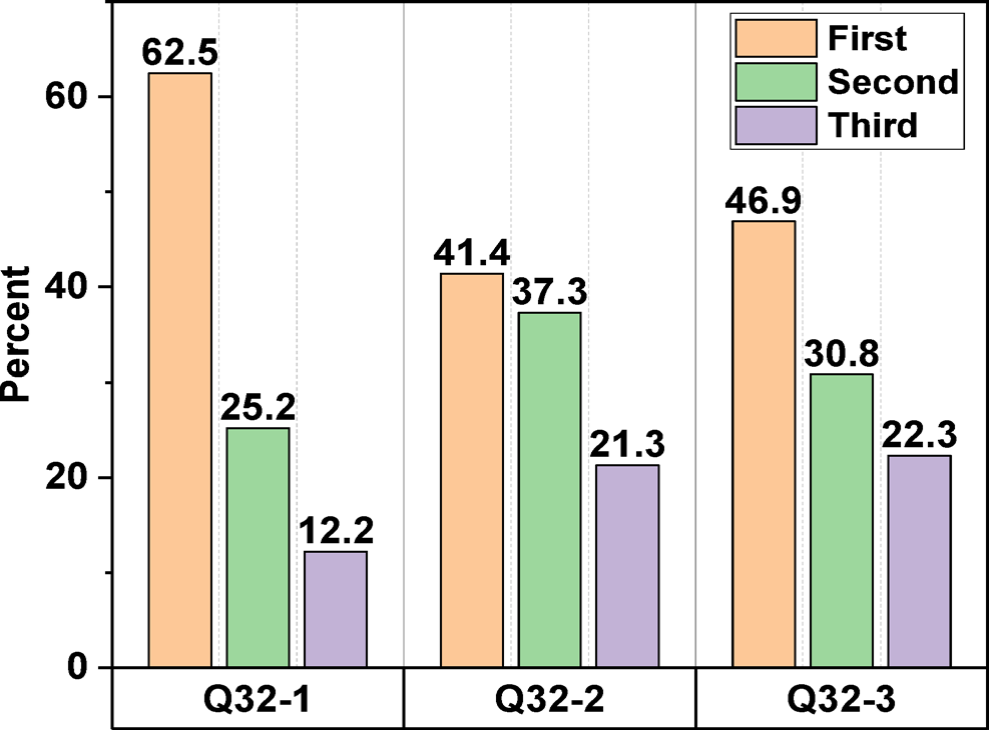
Participants’ response to question 32.

The level of physical contact appears to be inversely correlated with favourable perceptions of using treated wastewater. The most popular uses were washing cars and flushing toilets, while the least popular was washing clothes and taking baths^78^. According to Table 3, a majority of respondents express support for reusing treated wastewater for several beneficial purposes, including decreasing the application of toxic chemical fertilisers (88.9%), dropping pollution (87.9%), environmental preservation (85.8%), and alleviating the strain on dwindling aquifer networks and costly seawater desalination unit (86.6%). In question 31 (Arrange these incentives, in your view, to promote the use of treated wastewater by the general population; the range from 1 to 4 is from highest to lowest). Here is the overall rating, as shown in Fig. 5: reducing cost (53.8%, 1), reducing environmental damage (54%, 1), relieving stress on alternative water supplies (43.2%, 1), and wastewater representing an additional water source (40.4%, 2). In question 32 (From your point of view, what is the single most significant obstacle to the general public’s willingness to utilise treated wastewater? with 1 being the highest and 3 the lowest). As shown in Fig. 6, the following is the overall ranking of barriers: the fear of transmission of infectious diseases (62.5%, 1), ethical considerations or cultural issues (46.9%, 1), and quality and performance standards (41.4%, 1). Similarly, Buyukkamaci and Alkan^73^ demonstrated that the public is primarily concerned about potential health risks associated with reclaimed water usage, regardless of the treatment level.

#### Demographic analysis of acceptance of treated wastewater reuse

To explore the impact of demographic variables on participants’ acceptance of treated wastewater reuse, statistical analysis was conducted using a t-test to examine gender differences, and one-way ANOVA was used to examine differences between groups based on age, income, and educational level. These tests support a deeper understanding of the behavioural patterns associated with public attitudes toward reuse and determine whether these characteristics significantly influence acceptance.

An independent-sample t-test was conducted to analyse the differences between males and females in their confidence levels in using treated water in various applications, such as irrigation, industrial and commercial uses, and achieving multiple objectives. The results showed statistically significant differences between the sexes (refer to Table 4), with females showing higher confidence levels than males in all areas studied, including industrial and commercial uses (t = -3.67, *p* = 0.000), achieving multiple objectives (t = -4.03, *p* = 0.000), and irrigation (t = -4.62, *p* = 0.000).). Based on the authors’ knowledge, the findings agree with the Abdelrahman, et al.^38^ in some items. However, our outcomes contrast with previous studies indicating that men typically exhibit greater acceptance of risky technologies^5,42,83^. Therefore, when examining individuals’ responses to recycled water, it is imperative to account for sex variations.

**Table 4:** T-test outcomes show sex alteration in trust of recycled wastewater utilised for various purposes.

Purpose	Male		Female		T-Test	df	Sig.
	Mean	SD	Mean	SD			
Industrial & commercial uses	14.75	5.31	16.56	5.76	-3.67**	505	0.000
Different goals	21.39	5.8	23.61	6.19	-4.03**	505	0.000
Irrigation	10.05	4.07	11.81	4.15	-4.62**	505	0.000

** for female.

Data from Table 1 was regrouped into three categories—age, education level, and income—to examine how these factors affected respondents’ opinions about increasing recycled wastewater utilisation for other usages (Table 5).

**Table 5:** Preparing demographic data for one-way ANOVA and Chi-square tests by regrouping it according to age, education level, and income.

Groups	Symbol	Demographic parameter					
		Age(a) (Years)		Educational level(e)		Income(i) (IQ)	
Group 1	G1	G1a	< 35	G1e	Diploma or less	G1i	300,000 to 900,000
Group 2	G2	G2a	35–50	G2e	Undergraduate	G1i	900,000 to 1200,000
Group 3	G3	G3a	>= 50	G3e	Postgraduate	G1i	1,200,000 or more than

In addition to the t-test, one-way ANOVA was employed to analyse the impact of age, income, and educational level on a participant’s tendency towards using reclaimed water for industry, irrigation, commerce, different purposes, and multiple goals, aiming to achieve the fields outlined in Table 6. For the fields examined (industrial and commercial uses, irrigation, and other purposes), the results revealed that some variables were statistically significant (*p* < 0.05), while others had no statistically significant effect (*p* > 0.05). For example, no statistically significant difference was found between them and income, except for the " following goals " category. There was a significant difference (*p* = 0.01*) between groups, indicating that the differences between groups were not random. Specifically, there was a significant mean difference ($\:-$ 0.73) between G1i and G3i for G1i and a significant mean difference ($\:-$1.05) between G2i and G3i for G2i. This indicates that individuals with lower to middle incomes are more receptive to the idea. These findings contradict those of previous studies^84–86^, which concluded that higher income is associated with higher levels of agreeableness. In contrast, other factors did not show any statistically significant effect (*p* > 0.05), meaning the mean differences between groups were insignificant. For instance, regarding the utilisation of treated wastewater for irrigation, there is no statistically significant relationship between age groups (*p* = 0.17) or educational levels (*p* = 0.36).

**Table 6:** Results from a one-way ANOVA technique summarising the effects of age, education level, and income on people’s attitudes regarding using recycled wastewater.

Question	Groups															
	Age(years)					Educations(level)						Income (IQ)				
	Ga	G1a	G2a	G3a	Sig.	Ge		G1e	G2e	G3e	Sig.	Gi	G1i	G2i	G3i	Sig.
How will you trust using treated wastewater for irrigation?	1				0.17	1					0.36	1				0.436
	2	1.02				2		-0.033				2	0.71			
	3	0.03	-0.99			3		0.68	0.72			3	-0.037	-0.75		
How confident with using treated wastewater for industry and commerce?	1				0.57	1					0.11	1				0.061
	2	0.59				2		1.03				2	1.11			
	3	-0.53	-1.133			3		1.09	0.057			3	-0.74	-1.84		
How confident with using treated wastewater for the following purposes?	1				0.4	1					0.4	1				0.134
	2	1.01				2		0.78				2	1.61			
	3	-0.33	-1.34			3		0.56	-0.23			3	-0.06	-1.68		
The extent to which you support the use of treated wastewater for achieving the following goals	1				0.648	1					0.49	1				0.01*
	2	0.33				2		-0.17				2	0.32			
	3	0.33	-0.004			3		0.31	0.48			3	-0.73*	-1.05*		

Interestingly, these results contradict previous studies Bennett, et al.^87^, Chen, et al.^88^ and Buyukkamaci and Alkan^73^, which suggest that younger participants tend to be more agreeable. In contrast, Probe Smith, et al.^89^ and Bruvold and Cook^90^ found that older participants were more agreeable. Additionally, there was no statistically significant difference between educational levels across all fields examined, a finding consistent with two large-scale studies conducted in Australia by Dolnicar, et al.^77^ and the United States of America by Haddad, et al.^91^.

The results of this section are in line with the second objective of the research, as they offer preliminary insights into how demographic characteristics such as income, education, and age may influence public attitudes toward wastewater reuse, although most differences were not statistically significant. Previous studies by Lahlou, et al.^92^, Dolnicar, et al.^77^, and Fielding, et al.^15^ indicate that higher education levels are often associated with greater acceptance of sustainable technologies, which is supported by the results of this study. Although age-related differences were not statistically significant in our analysis, previous studies by Bennett, et al.^87^ and Buyukkamaci and Alkan^73^ have identified older populations as potentially less receptive to recycled water use. Therefore, awareness programs may still benefit from being tailored to address the specific concerns of older age groups.

#### Chi-square test

**Fig. 7:**
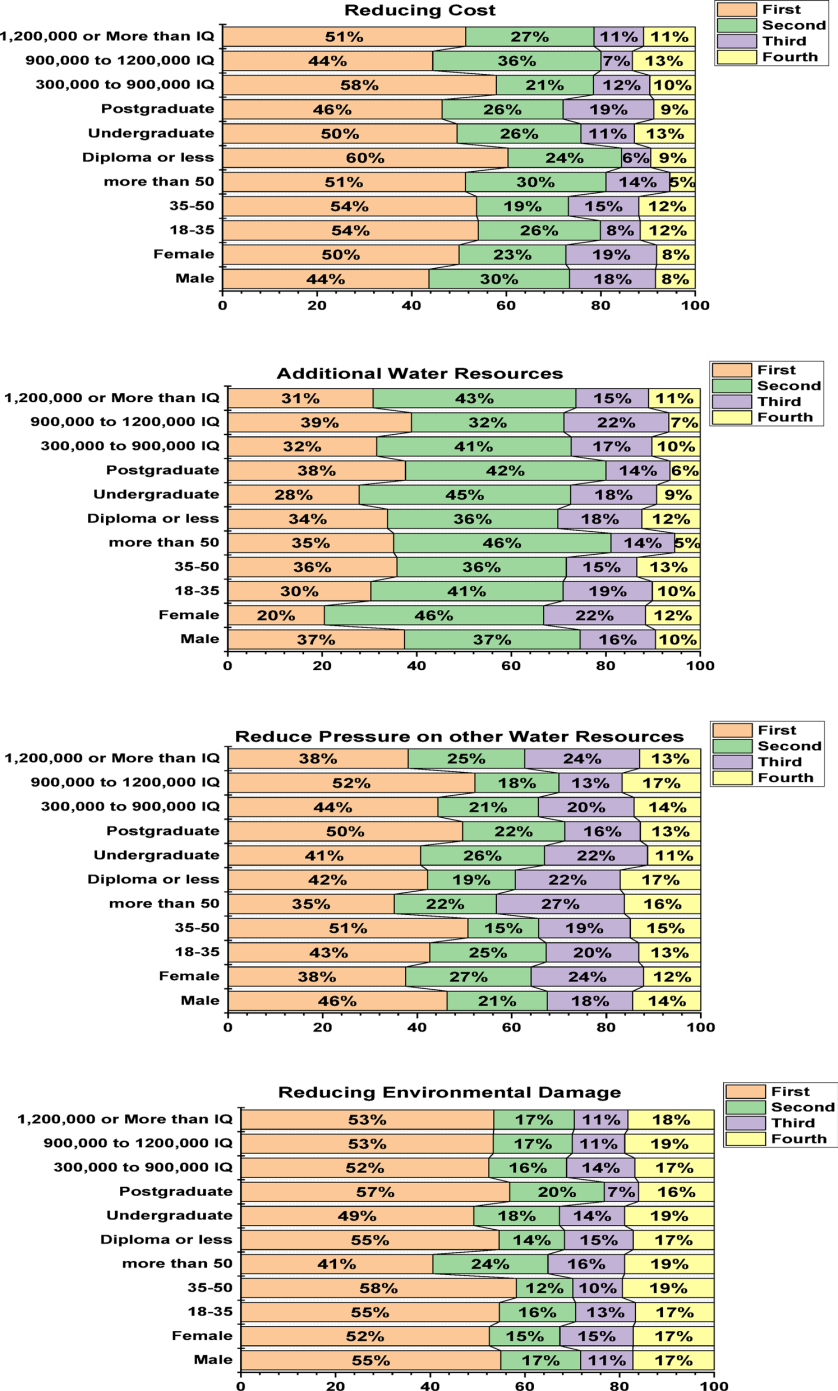
Chi-square analysis outcomes of the factors influencing the ranking of incentives to increase the reuse of recovered water.

A chi-square test was employed to analyse the respondents’ attitudes regarding the ranking of attitudes for treated wastewater reuse, based on demographic variables such as sex, age, education level, and income. Figure 7 illustrates that the top-ranked incentive for treated wastewater reuse is “cost reduction” was the most preferred motive among participants, being chosen as the first option by high percentages in multiple categories, including individuals with low education (60%), low income (58%), the 18–50 age group (54%), and females (50%), making it the highest ranked motive in terms of priority in the decision to support reuse of treated wastewater. Also, “providing an additional water source” was the most frequently selected motive as a second choice among participants, including females (46%), the over 50 age group (46%), middle education (undergraduates) (45%), and high income (43%), making it the second-ranked motive in terms of priority in the decision to support reuse of treated wastewater. Moreover, " reduced pressure on other water resources " was the most frequently selected motive as a third choice among participants, including individuals over 50 age group (27%), females (24%), and high income (24%), and low to middle education (22%), making it the third-ranked motive in terms of priority in the decision to support reuse of treated wastewater. Furthermore, " dropping environmental damage” was the most frequently selected motive as a fourth choice among participants, including individuals equal and over 35 age group (19%), middle income (19%), middle education (19%), and both male and female (17%), making it the fourth-ranked motive in terms of priority in the decision to support reuse of treated wastewater. It should be noted that the incentive ranking was determined based on the percentage of each motivation being selected as “first choice” only, without using weighted averages, as first choice is the most significant indicator of motivation priority for participants.

The results of the figure indicate that “cost reduction” is the most prioritised motivation among participants, reflecting a clear practical awareness of the importance of the economic aspect, making it the primary driver in the decision to support treated wastewater reuse. In second place, “providing an additional water source” emerged as a popular choice among participants, reflecting a growing strategic awareness of the importance of diversifying water resources and enhancing water security. “Reducing pressure on other water resources” was the third preferred choice, indicating considerable environmental awareness, albeit not a top priority. “Preserving the environment” came in fourth place, reflecting a general awareness of the importance of the environmental dimension, but it did not represent a decisive factor in decision-making.

**Fig. 8:**
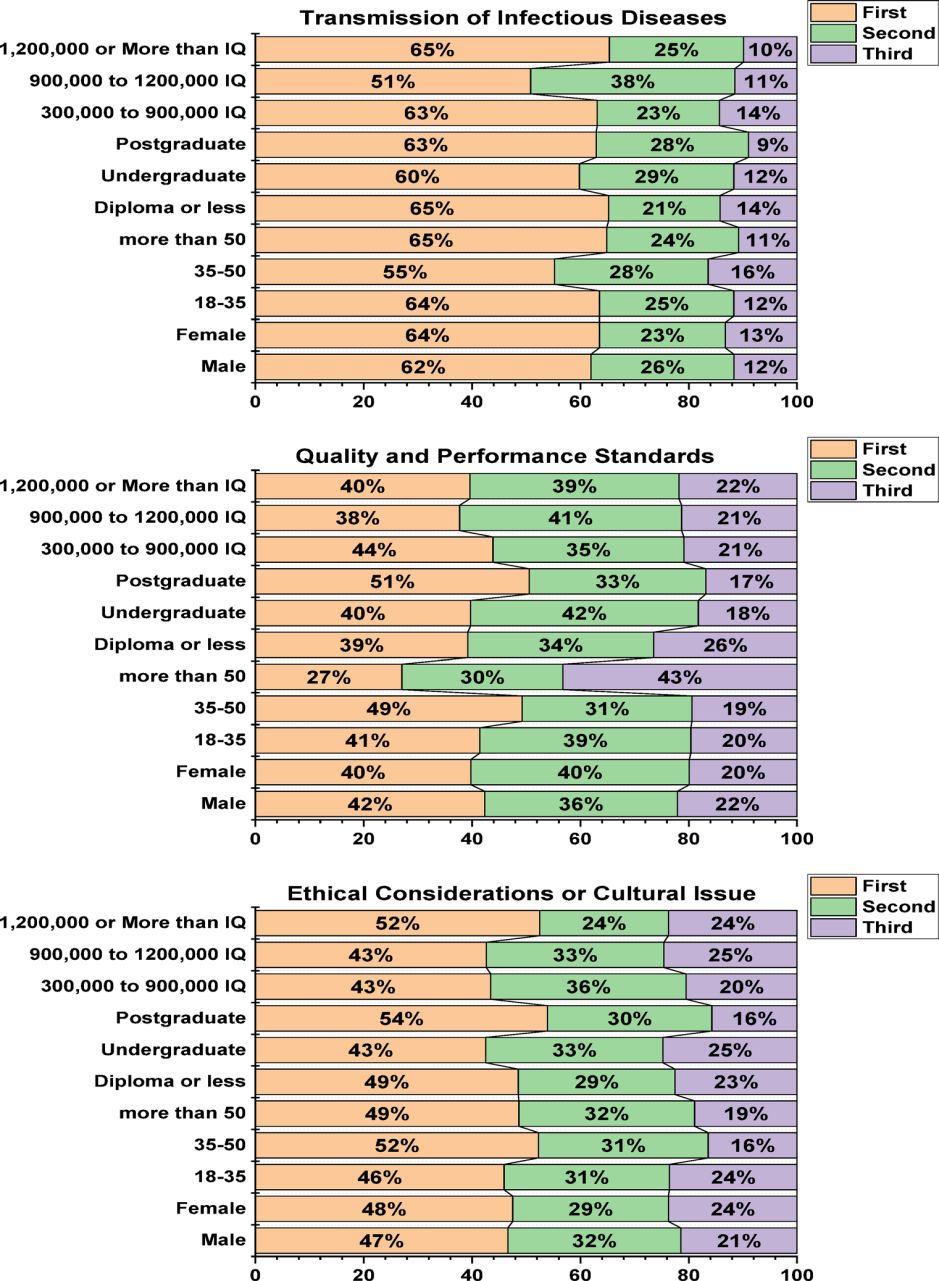
Chi-square analysis outcomes of the factors influencing the ranking barriers against expansion of recovered water.

Using a chi-square test, the study examined how respondents’ sex, age, education level, and income influenced the ranking of barriers to using treated wastewater. This analysis allowed us to understand how respondents rated these obstacles based on demographic variables such as sex, age, education level, and income. Figure 8 displays that “transmission of infectious diseases” is the most significant and influential barrier to refusal or hesitation among participants regarding the use of treated wastewater, with a very high percentage of respondents choosing it as their first choice across all demographic groups. The individuals with high income (65%), low education (65%), more than 50 aged (65%), and female (64%), making it clearly top the list of barriers and represents the primary obstacle to the acceptance of treated water for most participants. Also, the figure shows that “quality and performance standards” was the second most preferred motivation among participants. The individuals with middle education (42%), middle income (41%), female (40%), and 18–35 age (39%) make it the second option in the list of barriers and represent an important obstacle, but not the most important, to the acceptance of treated water for most participants. Moreover, the data shows that “ethical or cultural considerations” was ranked third. The individuals with middle income (25%), middle education (25%), female (24%), and 18–35 age (24%) make it ranked third among the obstacles, indicating that it is the least influential factor in shaping participants’ attitudes towards the use of treated water.

These findings of this section align with the third objective of the research, as they provide an analysis of the factors that promote or hinder treated water reuse. Previous studies by Shafiquzzaman, et al.^47^, Ablo and Etale^93^, and Lahlou, et al.^94^ confirm that health concerns are one of the most significant barriers to treated water reuse in Middle Eastern countries. Based on these findings, health awareness campaigns that explain water treatment processes and ensure their quality can increase population acceptance and reduce associated concerns.

The attitudes of inhabitants towards reusing treated wastewater can be better understood by combining the findings of the chi-square test with ANOVA. While chi-square finds the correlation between demographics and categorical orientations, ANOVA checks for statistical significance to make the findings more trustworthy. For instance, the ANOVA results showed that there was a statistical difference in confidence levels between the sexes, while the chi-square results showed that women were more likely to rate “reducing environmental damage” as the most important reason for reusing treated wastewater.

## Conclusion

The results of this study represent an important first step toward understanding public attitudes in Iraq toward treated wastewater reuse. Through a case study in Kut City, demographic variations in acceptance were highlighted, highlighting the pivotal role of economic and health factors in shaping participants’ attitudes. The results show that certain groups, such as women, the elderly, and those with limited income, demonstrated higher levels of reservations toward reuse, particularly regarding health risks and procedural aspects, such as the potential for disease transmission or doubts about the quality of treatment. This is attributed to the fact that women and the elderly are often more aware of potential health risks, while those with limited incomes fluctuate between the economic need to reduce costs, concerns about water safety, and their trust in responsible authorities. Accordingly, it is recommended that public policies and awareness programs prioritise these groups by enhancing institutional transparency and providing reliable health information, thus contributing to narrowing the trust gap and increasing community acceptance of treated wastewater use. The findings also emphasise the importance of aligning policy design with local populations’ social and economic characteristics to ensure greater effectiveness in adopting sustainable water management solutions.

In achieving the three research objectives, the results revealed the following: First, most participants expressed awareness of the water resource crisis, but demonstrated a lack of awareness of government measures, reflecting a knowledge gap that calls for institutional media interventions. Second, the data revealed a significant influence of gender in shaping participants’ attitudes toward using treated water, while differences in age and income were limited in certain areas only. Third, the primary motivation was cost reduction, particularly for those with limited income, while fear of transmitting infectious diseases was the most prominent barrier, reflecting an overlap between economic motivations and health concerns in shaping public attitudes.

Overall, this study sheds light on the social, economic, and psychological dimensions that influence Kut City society’s acceptance of the reuse of treated wastewater. The findings will contribute to developing water policies that are more responsive to target groups. This study also serves as a starting point for future studies covering broader regions and multiple research methods, enhancing water resource management’s effectiveness amidst growing challenges in Iraq.

## Limitations and future directions

The sample size is one of the methodological limitations of this study. Data was obtained from 507 participants in Kut city, meaning that not all Iraq perspectives may have been accurately represented. Additionally, response biases could have impacted the results; for example, certain participants might have had a greater familiarity with or interest in treated water reuse than others. Furthermore, while many statistical checks were conducted to guarantee the reliability of the analysis, additional research is needed to fully comprehend the significant influence of certain socioeconomic variables on the adoption of treated water reuse. Considering the study’s regional context, it can be compared to others that have looked at acceptance levels in Middle Eastern nations, including Turkey, Oman, and the United Arab Emirates. In those countries, acceptance levels vary according to cultural, religious, and economic reasons. Future research should take into consideration the fact that direct comparisons are complicated due to variations in infrastructure and government policy. The incentives and barriers categories were limited to a pre-defined list of options. Although they were based on previous studies, the lack of open-ended answers may have prevented the identification of other important motivations or concerns not included in the survey options. Similarly, the barriers analysed were limited to only three main options. While this structure provided valuable analytical information, it may have overlooked other influential factors such as aesthetics, lack of trust in responsible authorities, or broader social norms. Including explicit governance-related elements, such as trust in responsible authorities or operational transparency, within the incentives and barriers categories would enhance the depth and interpretability of the survey. Although data on religious affiliation were collected, the current study did not analyse this variable. Future studies are recommended to explore the potential influence of religion and related cultural norms on public attitudes, particularly in social contexts where these variables can play a critical role in shaping perceptions toward wastewater reuse.

Based on these limitations, this research recommends future studies that include a broader geographic scope and use qualitative research methods such as in-depth interviews to gain a deeper understanding of audience motivations. Also, the study made other recommendations to maximise water reuse’s societal acceptability: raising awareness of the water scarcity crisis in Iraq and promoting wastewater reuse initiatives. Recruit the help of psychology and water science specialists to spread the benefits of wastewater reuse that could have a positive financial impact on the country, its citizens, and the environment through online platforms, social media, and television. This study can be used as a springboard for further research in other Iraqi Governorates. In addition, future directions could include designing more flexible measurement tools that include open-ended questions or multidimensional options, allowing for the detection of indirect perceptions that closed-ended questionnaires may not capture. It is also recommended to expand the scope of the analysis to include variables that have not yet been implemented, such as religious dimensions or trust in institutions, within comprehensive explanatory models that help build a multidimensional understanding of public acceptance of water reuse projects in local contexts.

## Policy implications

In order to achieve water sustainability in Iraq, this study’s empirical findings can be used to propose a set of policy recommendations that will increase the acceptance and reuse of treated wastewater.

1. Public Awareness and Education Campaigns: Our findings indicate that 41% of respondents were unaware of Iraq’s water crisis, and 30% had never encountered the concept of wastewater recycling. This highlights the urgent need for targeted public education programs to increase awareness and acceptance of treated wastewater reuse. Accordingly, government agencies should collaborate with educational institutions, environmental NGOs, and social media platforms to develop campaigns that highlight the benefits of wastewater reuse while addressing common misconceptions (e.g., health risks and cultural concerns). This recommendation aligns with global success stories, such as Singapore’s “NEWater” initiative, which improved public confidence in water reuse through strategic communication and engagement.
2. Regulatory and Safety Standards Enforcement: While 62% of Iraqis trust existing wastewater treatment safety standards, concerns about quality and performance standards ranked as the second-largest barrier to adoption. Establishing stricter quality assurance frameworks, increasing transparency in water treatment processes, and conducting regular independent audits can enhance public trust in reclaimed water. Lessons from Australia and California show that public confidence improves when governments publish real-time water quality data and enforce clear safety benchmarks.
3. Financial Incentives to Drive Adoption: Our study revealed that cost reduction was the top motivation for wastewater reuse. Furthermore, 41% of participants were willing to pay extra fees for centralised treatment systems, suggesting an opportunity for incentive-based policy interventions. Implement tiered pricing models that offer reduced water tariffs for households using reclaimed water. Introduce subsidies or tax incentives for industries and farms that integrate treated wastewater into their operations. Similar policies in the UAE have successfully encouraged wastewater reuse by providing economic benefits to early adopters.
4. Infrastructure Investment and Decentralised Treatment Options: Our results show that 75% of respondents support installing centralised wastewater treatment systems but highlighted concerns about implementation costs and accessibility. Expand public-private partnerships (PPPs) to fund the development of wastewater treatment plants. Encourage localised, decentralised treatment systems in rural or underserved areas to reduce infrastructure costs and improve water security. Decentralised treatment models have been successfully implemented in Germany and India, reducing dependency on large-scale infrastructure while ensuring equitable water access.
5. Addressing the fear of disease transmission: The study results showed that fear of infectious disease transmission is the most significant barrier to accepting wastewater reuse. To address this barrier, it is recommended that awareness and educational campaigns be implemented that highlight advanced treatment technologies and health safety standards that ensure water is free of contaminants and health risks. Public confidence must also be enhanced through transparency, regular publication of quality test results, and the involvement of official health authorities in documenting the safety of treated water.

## Supplementary Information

Below is the link to the electronic supplementary material.

Supplementary Material 1

Supplementary Material 2

## Data Availability

The datasets used and/or analysed during the current study are available from the corresponding author upon reasonable request.
