# Supplementary material for: Examining the perceptions and permissions of reusing treated wastewater in a region facing water scarcity
Source: Sci Rep. 2025 Nov 18;15:40562. doi: 10.1038/s41598-025-24308-w (PMC12627639; doi:10.1038/s41598-025-24308-w)
Supplement: Supplementary file 1 — Supplementary Material 1 [file 41598_2025_24308_MOESM1_ESM.docx]

| Section 1: Demographic information | | | | | | | | | | | | | | | | | | | | | | |
| --- | --- | --- | --- | --- | --- | --- | --- | --- | --- | --- | --- | --- | --- | --- | --- | --- | --- | --- | --- | --- | --- | --- |
| Q1 | **Name** | | Optional | | | | | | | |  | | | | | | | | | | | |
| Q2 | **Sex** | | - Male | | | | | | | | - Female | | | | | | | | | | | |
| Q3 | **What is your age?** | |  | | | | | | | | | | | | | | | | | | | |
| Q4 | **Marital Status** | | - Married | | | | | | | | - Unmarried | | | | | - Divorced | | | - Widowed/Widow | | | |
| Q5 | **Place of residence (governorate)** | | |  | | | | | | | | | | | | | | | | | | |
| Q6 | **Religion** | - Muslim | | | | | - Christian | | | | | | | | | | - Other | | | | | |
| Q7 | **Occupation** | - Doctor | | | - Engineer | | | | | | | - Lawyer | | | | | | - Government employee | | | | |
|  |  | - Teacher | | | - Private employer | | | | | | | - Student | | | | | | - Other | | | | |
| Q8 | **Qualification** | - Primary | | | - Intermediate | | | | | - Secondary | | | | - Diploma | | | | - Undergraduate | | | - Postgraduate | |
| Q9 | **Are there any children living with you at home?** | | | | | | | | | - Yes | | | | | | | | - No | | | | |
| Q10 | **If your answer to question (9) is ” yes”, how many children are there ?** | | | | | | | - From 1 to 3 | | | | | | - From 4 to 6 | | | | - From 7 to 9 | | | | - > 9 |
| Q11 | **What is your monthly salary in Iraqi dinars?** | | - From 300,000 to 600,000 Iraqi dinars | | | | | | | | | | - From 600,000 to 900,000 Iraqi dinars | | | | | | | | | |
|  |  |  | - From 900,000 to 1,200,000 Iraqi dinars | | | | | | | | | | - From 1,200,000 to 1,500,000 Iraqi dinars | | | | | | | - More than 1,500,000 Iraqi dinars | | |
| Q12 | **Are you paying for municipal water consumption?** | | | | | - Yes | | | | | | | No | | | | | | | | | |
| Q13 | **How much money you pay for municipal water consumption in your home per month in Iraqi dinars?** | | | | | | | | | | | |  | | | | | | | | | |
| Q14 | **Is your home connected to the main sewerage network?** | | | | | | | | - Yes | | | | | | - No | | | | | | | |

| Section 2: Participants’ awareness of water resources and wastewater reuse | | | | | | | | |
| --- | --- | --- | --- | --- | --- | --- | --- | --- |
| Q15 | **Do you have any knowledge about water available in your country?** | - Yes | - No | | | | - I don’t know | |
| Q15-1 | **If your response to question (15) is "yes",**  **In your opinion, does Iraq have an inadequate supply of natural water resources ?** | - Yes | - No | | | | - I don’t know | |
| Q16 | **Is it necessary to reduce water consumption in Iraq, in your opinion ?** | - Yes | - No | | | | - I don’t know | |
| Q17 | **Do you take any measures to conserve water in your household use?** | - Yes | No | | | | | |
| Q18 | **If your response to question (17) is "yes", answer the following:** |  | | | | | | |
| Q18-1 | **Replaced all devices and fixtures with water saving models** | - Always | - Sometimes | | | | - Never | |
| Q18-2 | **Replaced all standard kitchen taps with economical taps** | - Always | - Sometimes | | | | - Never | |
| Q18-3 | **Water saving (e.g. when cleaning vegetables - bathing ..........)** | - Always | - Sometimes | | | | - Never | |
| Q18-4 | **Replaced traditional toilets with modern models that consume less water** | - Always | - Sometimes | | | | - Never | |
| Q19 | **Do you have any background knowledge on treated wastewater ?** | - Very familiar | | - Familiar | | | | - Unfamiliar |
|  |  | - Very unfamiliar | | | | I don’t know | | |
| Q20 | **What sources have you used to learn about treated wastewater reuse ?** | - TV | | | - Internet | | | - Journal |
|  |  | - Environmental groups | | | - Friend | | | - Family |
| Q21 | **From your different backgrounds, how would you suggest the communication with the public to be when implementing this type of project**? | |  | | | | | |

| Section 3: Respondent’s attitude towards the reuse of treated wastewater | | | | | | |
| --- | --- | --- | --- | --- | --- | --- |
| Q22 | **Are you in favour of using recycled wastewater for some purposes ?** | - I strongly agree | - I agree | | - I don’t agree | |
|  |  | - I refuse | - I strongly refuse | | | |
| Q23 | **Would you be open to having a central system installed in your home ?** | - Yes | - No | | - May be | |
| Q24 | **If your answer for question (23) is "yes or maybe", how much the extra monthly fees will you be willing to pay?** | |  | | | |
| Q25 | **How will you trust the safety criteria of treated wastewater that you can use?** | - Fully trust | - Trustworthy | | | - Trust it a bit |
|  |  | - Do not trust it at all | - Have no knowledge of it | | | |
| Q26 | **Are you willingness to participate in programs that support the use of treated wastewater?** | - Yes | - No | - May be | | |

| Section 4: Respondent’s trust in usage of treated wastewater for different purposes | | | | | | | | |
| --- | --- | --- | --- | --- | --- | --- | --- | --- |
| **Q27** | **How will you trust using treated wastewater for irrigation?** | | | | | | | |
| Q27-1 | Irrigation of food crops | - Strongly support | | - Support | - Don’t support | | | - I don’t know |
| Q27-2 | Irrigation of non-food crops | - Strongly support | | - Support | - Don’t support | | | - I don’t know |
| Q27-3 | Irrigation of public parks | - Strongly support | | - Support | - Don’t support | | | - I don’t know |
| Q27-4 | Animal crops | - Strongly support | | - Support | - Don’t support | | | - I don’t know |
| Q27-5 | Irrigation All crops | - Strongly support | | - Support | - Don’t support | | | - I don’t know |
| **Q28** | **How confident with using treated wastewater for industry and commerce?** | | | | | | | |
| Q28-1 | Cooling (e.g. cooling of power plants - cooling machines ....) | - Strongly support | - Support | | | - Don’t support | | - I don’t know |
| Q28-2 | Construction work (such as: construction work - mixing concrete...) | - Strongly support | - Support | | | - Don’t support | | - I don’t know |
| Q28-3 | Power plants (e.g. steam production ..............) | - Strongly support | - Support | | | - Don’t support | | - I don’t know |
| Q28-4 | Car wash | - Strongly support | - Support | | | - Don’t support | | - I don’t know |
| Q28-5 | Clothes Laundries | - Strongly support | - Support | | | - Don’t support | | - I don’t know |
| Q28-6 | Cleaning (street cleaning - cleaning workshops and garages - cleaning poultry farms .........) | - Strongly support | - Support | | | - Don’t support | | - I don’t know |
| Q28-7 | Fire Fighting | - Strongly support | - Support | | | - Don’t support | | - I don’t know |
| **Q29** | **How confident with using treated wastewater for the following purposes?** | | | | | | | |
| Q29-1 | Artificial lakes | - Strongly Support | - Support | | | | - Don’t support | - I don’t know |
| Q29-2 | Swimming Pools | - Strongly Support | - Support | | | | - Don’t support | - I don’t know |
| Q29-3 | Fish Farms | - Strongly Support | - Support | | | | - Don’t support | - I don’t know |
| Q29-4 | Household uses (e.g. bathing .............) | - Strongly Support | - Support | | | | - Don’t support | - I don’t know |
| Q29-5 | Storage for emergency | - Strongly Support | - Support | | | | - Don’t support | - I don’t know |
| Q29-6 | Drinking animals and birds | - Strongly Support | - Support | | | | - Don’t support | - I don’t know |
| Q29-7 | Washing vegetables and fruits | - Strongly Support | - Support | | | | - Don’t support | - I don’t know |
| Q29-8 | Cooking | - Strongly Support | - Support | | | | - Don’t support | - I don’t know |
| Q29-9 | Flushing toilet | - Strongly Support | - Support | | | | - Don’t support | - I don’t know |
| **Q30** | **The extent to which you support the use of treated wastewater for achieving the following goals** | | | | | | | |
| Q30-1 | Preserving the environment | - strongly support | | - support | | - Don’t support | | - I don’t know |
| Q30-2 | Easing pressure on groundwater and high-cost desalinated water | - strongly support | | - support | | - Don’t support | | - I don’t know |
| Q30-3 | Reducing pollution | - strongly support | | - support | | - Don’t support | | - I don’t know |
| Q30-4 | Giving up the purchase of chemical fertilizers harmful to Public health | - strongly support | | - support | | - Don’t support | | - I don’t know |

| Section 5: Participants’ opinion regarding the incentive for encouraging the public to reuse treated wastewater and the barriers preventing public from reusing treated wastewater | | | | | |
| --- | --- | --- | --- | --- | --- |
| **31** | **Arrange these incentives, in your view, to promote the use of treated wastewater by the general population (1 is the highest and 4 is the lowest)** | | | | |
| Q31-1 | Reduce cost | - First | - Second | - Third | - Fourth |
| Q31-2 | Additional water resources | - First | - Second | - Third | - Fourth |
| Q31-3 | Reduce pressure on other water resources | - First | - Second | - Third | - Fourth |
| Q31-4 | Reducing environmental damage | - First | - Second | - Third | - Fourth |
| **Q32** | **From your point of view, what is the single most significant obstacle to the general public's willingness to utilise treated wastewater ? (1 is the highest and 3 is the lowest)** | | | | |
| Q32-1 | Transmission of infectious diseases | - First | - Second | - Third |  |
| Q32-2 | Quality and performance standards | - First | - Second | - Third |  |
| Q32-3 | Ethical considerations or cultural issue | - First | - Second | - Third |  |
