# Supplementary material for: Examining the perceptions and permissions of reusing treated wastewater in a region facing water scarcity
Source: Sci Rep. 2025 Nov 18;15:40562. doi: 10.1038/s41598-025-24308-w (PMC12627639; doi:10.1038/s41598-025-24308-w)
Supplement: Supplementary file 2 — Supplementary Material 2 [file 41598_2025_24308_MOESM2_ESM.docx]

| **استبانة حول اعادة استخدام المياه** | | | | | | | | | | | | |
| --- | --- | --- | --- | --- | --- | --- | --- | --- | --- | --- | --- | --- |
| **القسم الأول:**  **المعلومات الديموغرافية** | | | | | | | | | | | | |
| **1** | **الاسم (اختياري)** |  | | | | | | | | | | |
| **2** | **الجنس** | - ذكر | - انثى | | | |  | | | | | |
| **3** | **العمر** |  | | | | | | | | | | |
| **4** | **الحالة الاجتماعية** | - متزوج | - أعزب | | | | - مطلق | | | | | - أرمل/ارملة |
| **5** | **مكان السكن** |  | | | | | | | | | | |
| **6** | **الديانة** | - مسلم | | - مسيحي | | | | - اخرى | | | |  |
| **7** | **المهنة** | - طبيب | | - مهندس | | | | - محامي | | | | - موظف |
|  |  | - مدرس | | - صاحب عمل خاص | | | | - طالب | | | | - اخرى |
| **8** | **المؤهل العلمي** | - ابتدائي | | - متوسطة | | | | - اعدادي | | | | - دبلوم |
|  |  | - بكالوريوس | | - دراسات عليا | | | |  | | | | |
| **9** | **هل هناك أطفال يعيشون معك في المنزل؟** | - نعم | | - لا | | | |  | | | | |
| **10** | **إذا كانت إجابتك على السؤال (9) بـ "نعم"، فكم عدد الأطفال هناك؟** | - من 1الى 3 | | - من 4 الى 6 | | | | - من 7 الى 9 | | | - أكثر من 9 | |
| **11** | **ما هو دخلك الشهري بالدينار العراقي؟** | - من 000, 300 الى 000 ,600 دينار عراقي | | | | - من 000 ,600 الى000 ,900 دينار عراقي | | | | | | |
|  |  | - من 000 ,900 الى000 ,1,200 دينار عراقي | | | | - من 1,200,000الى 1,500,000 دينار عراقي | | | | - أكثر من 1,500,000 دينار عراقي | | |
| **12** | **هل تدفع ثمن استهلاك المياه الذي تستخدمه في منزلك (مياه الاسالة)** | | | | - نعم | | | - لا |  | | | |
| **13** | **كم تدفع شهريًا مقابل استهلاك المياه المستخدمة في منزلك بالدينار العراقي؟** | | | | | | |  | | | | |
| **14** | **هل منزلك مرتبط بشبكة الصرف الصحي الرئيسية؟** | | | | - نعم | | | - لا | | | | |

|  | **(القسم الثاني): وعي المشاركين بموارد المياه وإعادة استخدام مياه الصرف الصحي** | | | | | |
| --- | --- | --- | --- | --- | --- | --- |
| **15** | **هل**  **لديك أي معلومات عن المياه المتوفرة في بلدك؟** | - نعم | - لا | | - لا اعرف | |
| **15-1** | **إذا كانت إجابتك على السؤال (15) بنعم، فبرأيك هل يعاني العراق من نقص في موارد المياه الطبيعية؟** | - نعم | - لا | | - لا اعرف | |
| **16** | **هل من الضروري تقليل استهلاك المياه في العراق، في رأيك؟** | - نعم | - لا | | - لا اعرف | |
| **17** | **هل تتخذ أي إجراءات للحفاظ على المياه في استخدامك المنزلي؟** | - نعم | - لا | | | |
| **18** | **إذا كانت إجابتك على سؤال (17) (بنعم)، فأجب عما يلي:** | | | | | |
|  | **1-استبدال جميع الأجهزة والتركيبات بنماذج موفرة للمياه** | - دائما | | - احيانا | | - ابدا |
|  | **2-استبدال جميع صنابير المطبخ القياسية بصنابير اقتصادية** | - دائما | | - احيانا | | - ابدا |
|  | **3-توفير الماء (مثلا عند تنظيف الخضار - الاستحمام ..........)** | - دائما | | - احيانا | | - ابدا |
|  | **4-استبدال المراحيض التقليدية بنماذج حديثة تستهلك كميات أقل من المياه** | - دائما | | - احيانا | | - ابدا |
| **19** | **هل لديك أي خلفية معرفية عن مياه الصرف الصحي المعالجة؟** | - معرفة قوية | | - معرفة بسيطة | | - غير مألوفة |
|  |  | - لا اعرف شيئا عنها | | - غير مألوف للغاية | | |
| **20** | **ما هي المصادر التي استخدمتها للتعرف على إعادة استخدام مياه الصرف الصحي المعالجة؟** | - التلفزيون | | - الانترنيت | | - المجلات |
|  |  | - المجاميع البيئية | | - الاصدقاء | | - العائلة |
| **21** | **من خلال خلفيتك المختلفة، كيف تقترح أن يتم التواصل مع الجمهور عند تنفيذ هذا النوع من المشاريع؟** |  | | | | |

|  | **(القسم الثالث):**  **موقف المستجيب اتجاه إعادة استخدام مياه الصرف الصحي المعالجة** | | | | | | | | | |  |
| --- | --- | --- | --- | --- | --- | --- | --- | --- | --- | --- | --- |
| **22** | **هل أنت مع استخدام مياه الصرف الصحي المعاد تدويرها لبعض الأغراض؟** | - اوافق بشدة | | - اوافق | - لا اوافق | | - ارفض | | | - ارفض بشدة |  |
| **23** | **هل ستكون منفتحًا على تركيب نظام مركزي في منزلك؟** | - نعم | | - لا | | | - ربما | | | | |
| **24** | **إذا كانت إجابتك على السؤال (23) هي "نعم أو ربما"، ما هو مقدار الرسوم الشهرية الإضافية التي ستكون على استعداد لدفعها؟** | |  | | | | | | | |  |
| **25** | **كيف ستثق بمعايير السلامة الخاصة بمياه الصرف الصحي المعالجة التي يمكنك استخدامها؟** | | - اثق به تماما | | | - جدير بالثقة | | - اثق به قليلا | | |  |
|  |  |  | - لا اثق به تماما | | | لا اعلم بها | | | | |  |
| **26** | **هل انت على استعداد للمشاركة في البرامج التي تدعم استخدام مياه الصرف الصحي المعالجة؟** | | - نعم | | | - لا | | | - ربما | |  |

| **(القسم الرابع): ثقة المستجيب في استخدام مياه الصرف الصحي المعالجة لأغراض مختلفة** | | | | | | |
| --- | --- | --- | --- | --- | --- | --- |
| **27** | **كيف ستثق في استخدام مياه الصرف الصحي المعالجة للري؟** | | | | | |
|  | 1-ري المحاصيل الغذائية | - اثق بشدة | | - اثق | - لا اثق | - لا اعرف |
|  | 2-لري المحاصيل غير الغذائية | - اثق بشدة | | - اثق | - لا اثق | - لا اعرف |
|  | 3-لري الحدائق العامة | - اثق بشدة | | - اثق | - لا اثق | - لا اعرف |
|  | 4- لري محاصيل علف الحيوانات | - اثق بشدة | | - اثق | - لا اثق | - لا اعرف |
|  | 5-لري جميع المحاصيل | - اثق بشدة | | - اثق | - لا اثق | - لا اعرف |
| **28** | **ما مدى** ثقتك **في استخدام مياه الصرف الصحي المعالجة في الصناعة والتجارة؟** | | | | | |
|  | 1-التبريد (مثلا تبريد محطات توليد الكهرباء –تبريد الأليات الصناعية ....) | - اثق بشدة | | - اثق | - لا اثق | - لا اعرف |
|  | 2-الأعمال الإنشائية (مثل: أعمال البناء – خلط الخرسانة…) | - اثق بشدة | | - اثق | - لا اثق | - لا اعرف |
|  | 3-محطات توليد الطاقة (مثل إنتاج البخار ..............) | - اثق بشدة | | - اثق | - لا اثق | - لا اعرف |
|  | 4- غسيل السيارات | - اثق بشدة | | - اثق | - لا اثق | - لا اعرف |
|  | 5-غسل الملابس | - اثق بشدة | | - اثق | - لا اثق | - لا اعرف |
|  | 6-التنظيف (تنظيف الشوارع - تنظيف الورش والكراجات -  تنظيف مزارع الدواجن....) | - اثق بشدة | | - اثق | - لا اثق | - لا اعرف |
|  | 7- مكافحة الحرائق | - اثق بشدة | | - اثق | - لا اثق | - لا اعرف |
| **29** | **ما مدى الثقة في استخدام مياه الصرف الصحي المعالجة للأغراض التالية؟** | | | | | |
|  | 1-بحيرات صناعية | - اثق بشدة | | - اثق | - لا اثق | - لا اعرف |
|  | 2- احواض السباحة | - اثق بشدة | | - اثق | - لا اثق | - لا اعرف |
|  | 3- بحيرات الاسماك | - اثق بشدة | | - اثق | - لا اثق | - لا اعرف |
|  | 4- الاستخدامات المنزلية (مثل الاستحمام .............) | - اثق بشدة | | - اثق | - لا اثق | - لا اعرف |
|  | 5-تخزين لحالات الطوارئ | - اثق بشدة | | - اثق | - لا اثق | - لا اعرف |
|  | 6-مياة شرب للحيوانات والطيور | - اثق بشدة | | - اثق | - لا اثق | - لا اعرف |
|  | 7-غسل الخضار والفواكه | - اثق بشدة | | - اثق | - لا اثق | - لا اعرف |
|  | 8- لغرض الطبخ | - اثق بشدة | | - اثق | - لا اثق | - لا اعرف |
|  | 9- تنظيف المرحاض | - اثق بشدة | | - اثق | - لا اثق | - لا اعرف |
| **30** | **مدى دعمكم لاستخدام المياه المعاد تدويرها لتحقيق الأهداف التالية:** | | | | | |
|  | 1-الحفاظ على البيئة | | - ادعم بقوة | - ادعم | - لا ادعم | - لا اعرف |
|  | 2-تخفيف الضغط على المياه الجوفية والمياه المحلاة ذات التكلفة العالية | | - ادعم بقوة | - ادعم | - لا ادعم | - لا اعرف |
|  | 3- الحد من التلوث | | - ادعم بقوة | - ادعم | - لا ادعم | - لا اعرف |
|  | 4- التخلي عن شراء الأسمدة الكيماوية الضارة بالصحة العامة | | - ادعم بقوة | - ادعم | - لا ادعم | - لا اعرف |

| **(القسم الخامس): رأي المشاركين في الحافز لتشجيع الجمهور على إعادة استخدام مياه الصرف الصحي المعالجة والحواجز التي تمنع الجمهور من إعادة استخدام مياه الصرف الصحي المعالجة** | | | | | |
| --- | --- | --- | --- | --- | --- |
| **31** | **رتب هذه الحوافز، من وجهة نظرك، لتشجيع استخدام مياه الصرف الصحي المعالجة من قبل عامة السكان (1 هو الأعلى و4 هو الأدنى):** | | | | |
|  | اولا-تقليل التكلفة | - 1 | - 2 | - 3 | - 4 |
|  | ثانيا-توفير مصادر مياه إضافية | - 1 | - 2 | - 3 | - 4 |
|  | ثالثا-تقليل الضغط على مصادر المياه الأخرى | - 1 | - 2 | - 3 | - 4 |
|  | رابعا- تقليل الأضرار البيئية | - 1 | - 2 | - 3 | - 4 |
| **32** | **من وجهة نظرك، ما هي العقبة الأكثر أهمية أمام رغبة عامة الناس في الاستفادة من مياه الصرف الصحي المعالجة؟ (1 هو الأعلى و3 هو الأدنى):** | | | | |
|  | اولا- نقل الامراض المعدية | - 1 | - 2 | - 3 | |
|  | ثانيا- معايير الجودة والأداء | - 1 | - 2 | - 3 | |
|  | ثالثا- اعتبارات أخلاقية أو قضية ثقافية | - 1 | - 2 | - 3 | |
